# Urinary Concentrations of Bisphenol A and Phthalate Metabolites Measured during Pregnancy and Risk of Preeclampsia

**DOI:** 10.1289/EHP188

**Published:** 2016-05-13

**Authors:** David E. Cantonwine, John D. Meeker, Kelly K. Ferguson, Bhramar Mukherjee, Russ Hauser, Thomas F. McElrath

**Affiliations:** 1 Division of Maternal-Fetal Medicine, Department of Obstetrics and Gynecology, Brigham and Women’s Hospital, Harvard Medical School, Boston, Massachusetts, USA; 2 Department of Environmental Health Sciences, and; 3 Department of Biostatistics, University of Michigan School of Public Health, Ann Arbor, Michigan, USA; 4 Department of Environmental Health, Harvard T.H. Chan School of Public Health, Boston, Massachusetts, USA

## Abstract

**Background:**

Preeclampsia represents a major cause of maternal mortality and morbidity worldwide. Although it is known that the placenta plays a central role in development of preeclampsia, investigation into the contribution of environmental toxicants to the risk of preeclampsia has been sparse.

**Objectives:**

In the present study we examined the relationship between longitudinally measured urinary BPA and phthalate metabolite concentrations during gestation and preeclampsia.

**Methods:**

A nested case–control study of preterm birth was performed in 2011 from women enrolled in a prospective birth cohort study at Brigham and Women’s Hospital in Boston. There were 50 cases of preeclampsia as part of this study. Urine samples were analyzed for concentrations of BPA and nine phthalate metabolites several times during pregnancy. Adjusted Cox proportional hazard models were used to calculate hazard ratios of preeclampsia in association with an interquartile range increase in BPA and phthalate concentrations and were weighted to reflect results generalizable to the base population.

**Results:**

Adjusted hazard ratios indicated that an interquartile range increase of urinary concentrations of BPA (1.53; 95% CI: 1.04, 2.25) and MEP (monoethyl phthalate) (1.72; 95% CI: 1.28, 2.30) at 10 weeks gestation was associated with onset of preeclampsia, whereas significantly elevated hazard ratios were found across gestation for all DEHP [di(2-ethylhexyl) phthalate] metabolites. These relationships differed based on infant sex.

**Conclusions:**

Urinary concentrations of BPA and several phthalate metabolites were significantly associated with increased risk of preeclampsia. If validated, these results indicate an environmental contribution of endocrine-disrupting chemicals to preeclampsia and suggest a modifiable means to reduce the mortality and morbidity associated with this condition.

**Citation:**

Cantonwine DE, Meeker JD, Ferguson KK, Mukherjee B, Hauser R, McElrath TF. 2016. Urinary concentrations of bisphenol A and phthalate metabolites measured during pregnancy and risk of preeclampsia. Environ Health Perspect 124:1651–1655; http://dx.doi.org/10.1289/EHP188

## Introduction

Preeclampsia (PE) is characterized by new-onset or worsening hypertension and significant proteinuria after 20 weeks of gestation and still represents a major cause of preterm birth and maternal mortality/morbidity worldwide (ACOG 2013; Ananth et al. 2013; Ilekis et al. 2007). Decreased smoking prevalence, increasing rates of obesity, chronic hypertension, and diabetes in the United States are all thought to partly explain the increasing trend, though other unknown behavioral, genetic, and environmental factors certainly play a role (Berg et al. 2009; Ogden et al. 2006; Catov et al. 2007).

Phthalates and bisphenol A (BPA) are two classes of human-made chemicals produced in high volume and used in an immense variety of products and applications worldwide. Higher molecular weight phthalates, such as di(2-ethylhexyl) phthalate (DEHP) and butylbenzl phthalate (BBzP), are most commonly used as plasticizers in a variety of polyvinyl chloride containing products, including medical materials, and exposure in humans primarily occurs from the consumption of contaminated food and water (NRC 2008; Pak et al. 2007; Schettler 2006; Wormuth et al. 2006). Lower-molecular-weight phthalates, such as diethyl phthalate (DEP) and dibutyl phthalate (DBP), are primarily used in solvents and adhesives which are found in a wide variety of consumer personal care products (Duty et al. 2005; NRC 2008). BPA is most commonly used in the production of epoxy resins and polycarbonate polymers and is found in a variety of consumer products such as food can linings, water bottles, thermal receipts, and water supply pipes. Both of these classes of chemicals are easily released into the environment, which results in ubiquitous exposures to the general population (Calafat et al. 2008; Silva et al. 2004).

Although the specific pathophysiology underlying PE remains unclear, it is known that PE is associated with abnormal placentation (McElrath et al. 2008; Redman and Sargent 2005). The presence of the placenta, and particularly trophoblastic cells, is necessary for the development of PE. Specifically, limited trophoblastic invasion of the maternal decidual spiral arteries has been called the “hallmark” of PE pathology (Redman and Sargent 2005). It has been demonstrated that BPA can affect the proliferative process of trophoblastic cells through estrogen-related receptor γ (ERRγ1) (Morice et al. 2011) and has a dose-dependent effect upon apoptosis of primary human cytotrophoblast cells via tumor necrosis factor α (Benachour and Aris 2009). Phthalates have been shown to affect placental gene expression (Adibi et al. 2010) in human placental tissue and alter activation of peroxisome proliferator–activated receptor γ (PPARγ) in rat placentas (Xu et al. 2008). These results demonstrate the potential for both of these toxicants to decrease placental growth and disturb function, providing plausible mechanisms for heightened risk of developing preeclampsia.

To our knowledge, there has been only one small epidemiological study investigating the relationship between BPA and preeclampsia (Leclerc et al. 2014) and no studies into the role of phthalate exposure and this heterogeneous condition. In the present study we examined the relationship between longitudinally measured urinary BPA and phthalate metabolite concentrations during gestation and risk of preeclampsia.

## Methods

### Study Population

Details of the parent birth cohort have been previously described (McElrath et al. 2012). Briefly, a total of 2,246 women were recruited from 2006 to 2008 at three tertiary care academic centers: Brigham and Women’s Hospital and Beth Israel Deaconess Medical Center in Boston, Massachusetts, and University of Pennsylvania in Philadelphia, Pennsylvania. In 2011, a nested case–control study of singleton preterm birth was selected from the Brigham and Women’s Hospital participant pool of women (*n* = 1,648) who were originally enrolled as part of this larger prospective birth cohort. These women were originally recruited at two Brigham and Women’s Hospital clinical facilities and one private practice facility. This nested case–control study of preterm birth consisted of 130 women who delivered at < 37 weeks of gestation and 352 randomly selected women who delivered at ≥ 37 weeks. Of the 482 women who were a part of the nested case–control study, a total of 50 (10.4%) were diagnosed with PE. Of the 50 cases of PE, 31 (62.0%) were diagnosed < 37 weeks gestation. The study was approved by the institutional review boards of Brigham and Women’s Hospital and the University of Michigan.

Maternal spot urine samples were obtained at four visits during pregnancy. Initial visit samples were collected at median 9.7 weeks gestation (range, 4.7–16.1 weeks), visit 2 at median 17.9 weeks (range, 14.9–21.9 weeks), visit 3 at median 26.0 weeks (range, 22.9–29.3 weeks), and visit 4 at median 35.1 weeks (range, 33.1–38.3 weeks). All specimens were stored at –80°C until analysis. Demographic information was collected at the initial visit. Clinically relevant pregnancy characteristics were collected at the initial visit and subsequently at three additional time points throughout pregnancy.

### Definition of Preeclampsia

Preeclampsia was defined as blood pressures ≥ 140 mmHg systolic or ≥ 90 mmHg diastolic after 20 weeks of gestation along with positive urinary protein testing (> 300 mg/24 hr or protein/creatinine ratio > 0.20). All cases of preeclampsia were de-identified and reviewed by a panel of the study principle investigators. A final diagnosis was assigned only with the approval of this panel.

### Urinary BPA and Phthalate Concentrations

Total BPA (free + conjugated) and nine phthalate metabolites [mono(2-ethylhexyl) phthalate (MEHP), mono(2-ethyl-5-hydroxyhexyl) phthalate (MEHHP), mono(2-ethyl-5-oxohexyl) phthalate (MEOHP), mono(2-ethyl-5-carboxypentyl) phthalate (MECPP), mono-benzyl phthalate (MBzP), Mono-*n*-butyl phthalate (MBP), mono-isobutyl phthalate (MiBP), mono-ethyl phthalate (MEP), mono(3-carboxypropyl) phthalate (MCPP)] were measured in all available urine samples (*n* = 1,695) by NSF International (Ann Arbor, MI), based on methods developed by the Centers for Disease Control (CDC) (Lewis et al. 2013; Silva et al. 2007; Ye et al. 2005). Levels below the limit of detection (LOD) were kept if a numerical value was reported or replaced by dividing the LOD by the square root of 2 if no value was reported (Hornung and Reed 1990). Urinary specific gravity (SG) was measured in all samples as an indicator of urine dilution using a digital handheld refractrometer (ATAGO Company Ltd., Tokyo, Japan). Urinary BPA and phthalate concentrations were corrected for SG using the following formula: *P_c_ = P*[(1.015 – 1)]/*SG* – 1)], where *P_c_* represents the SG-corrected BPA concentration (nanograms per milliliter), *P* represents the measured concentration in urine, 1.015 is the median SG of all samples measured, and *SG* represents the SG of the individual sample (Meeker et al. 2009). Both uncorrected and SG-corrected metabolite levels were log-normally distributed and were natural log (ln) transformed for statistical analysis to more closely approximate normality and to reduce the likelihood of influential values given the skewed distribution.

### Statistical Analysis

Analysis was performed using SAS version 9.4 (SAS Institute Inc., Cary, NC). *p*-Values < 0.05 were used to define statistical significance. Sociodemographic characteristics of the participating women were described and associations between those with and without PE were examined using chi-square, Fisher’s exact, or Wilcoxon rank-sum test as appropriate. Geometric means and standard deviations of SG-corrected BPA and phthalate levels at individual visits were calculated, and differences by visits between cases and controls of preeclampsia were tested using Wilcoxon rank-sum test. For all statistical analyses, we used inverse probability weightings created from the probability of selection from the parent study population for cases (90.1%) and controls (33.9%) (Ferguson et al. 2015b). This adjustment negates the effect of oversampling preterm births and makes results generalizable to pregnant women in the base Brigham and Women’s Hospital cohort population (Jiang et al. 2006).

Initially, geometric average BPA and phthalate concentrations were calculated using the visit 1–visit 3 time point measurements and used in separate Cox proportional hazard regression models where PE was the outcome. Visit 4 measurements were excluded from the average due to a disproportionate number of samples available in preterm cases compared to controls from that time point. Crude models included average urinary SG as a covariate. In full models, maternal age, race/ethnicity (white/African American/other), and prepregnancy body mass index (BMI) were included *a priori*, and additional covariates were added in a forward step-wise model selection procedure with inclusion in final models if they altered effect estimates by > 10%. Additional variables that were considered included health insurance category (private/HMO/self-pay vs. Medicaid/Supplemental Security income/MassHealth), maternal education, smoking status during pregnancy (yes/no), parity (nulliparous/parous), gestational diabetes (yes/no), prior history of PE (yes/no), and use of assisted reproductive technology (ART) (yes/no). Windows of vulnerability to BPA or phthalate exposure were then assessed by fitting separate Cox proportional hazard regression models with PE as the outcome to calculate hazard ratios corresponding to an interquartile range (IQR) increase in urinary BPA or phthalate metabolite levels from each individual visit. We additionally ran models of PE stratified by infant sex and by gestational age at disease onset, and as a sensitivity analysis we excluded cases of superimposed PE.

## Results

Demographic and clinical characteristics of the total study population and stratified by preeclampsia diagnosis are presented in Table 1. In general, our population had a mean ± SD age of 32.1 ± 5.4 years, had a BMI of 26.3 ± 6.1 kg/m^2^, were largely Caucasian (60.0%), and were highly educated (85.6% with post–high school education). Cases of preeclampsia in this population had a significantly higher BMI at initial visit (31.1 ± 7.6 vs. 25.7 ± 5.6 kg/m^2^; *p* < 0.001), used ART for conception (18.0% vs. 8.4%; *p* = 0.04), had gestational diabetes (18.0% vs. 7.2%; *p* = 0.03), and were previously diagnosed with chronic hypertension (32.0% vs. 3.7%; *p* < 0.001). Additionally, cases of preeclampsia were born earlier (36.1 vs. 38.1 weeks of gestation; *p* < 0.001), weighed less at delivery (2683.9 vs. 3174.3 g; *p* < 0.001), and were more likely to be female (60.0% vs. 42.5%; *p* = 0.02).

**Table 1:** Weighted baseline characteristics of preeclampsia (PE) diagnosed pregnancies compared to all other pregnancies [mean ± SD or *n* (%)].

Characteristic	Total (*n* = 481)	PE (*n* = 50)	No PE (*n* = 431)	*p*-Value^*a*^
Age (years)	32.1 ± 5.4	32.7 ± 5.8	32.1 ± 5.4	0.72
BMI at initial visit (kg/m^2^)	26.3 ± 6.1	31.1 ± 7.6	25.7 ± 5.6	< 0.001
Race
White	281 (58.4)	30 (60.0)	251 (58.2)	0.18
African American	77 (16.0)	13 (26.0)	64 (14.9)
Asian	31 (6.4)	1 (2.0)	30 (7.0)
Hispanic	66 (13.7)	4 (8.0)	62 (14.4)
Other	26 (5.4)	2 (4.0)	24 (5.6)
Maternal education (years)^*b*^
< 12	18 (3.8)	2 (4.0)	16 (3.8)	0.25
High school/GED equivalent	50 (10.6)	9 (18.0)	41 (9.8)
> 12	403 (85.6)	39 (78.0)	363 (86.4)
Health insurance^*c*^
Self-pay or Medicaid/Mass Health	91 (19.4)	10 (20.0)	81 (19.3)	0.85
Private insurance/HMO	379 (80.6)	40 (80.0)	339 (80.7)
Nulliparous	215 (44.7)	26 (52.0)	189 (43.9)	0.33
Smoked during pregnancy	15 (3.1)	4 (8.0)	11 (2.6)	0.06
Use of assisted reproductive technology	45 (9.4)	9 (18.0)	36 (8.4)	0.04
Familial history of type 2 diabetes	221 (45.9)	29 (58.0)	192 (44.6)	0.10
Current diagnosis of gestational diabetes	40 (8.3)	9 (18.0)	31 (7.2)	0.03
Preeclampsia in previous pregnancy	17 (3.5)	9 (18.0)	8 (2.0)	< 0.001
History of chronic hypertension	32 (6.7)	16 (32.0)	16 (3.7)	< 0.001
Gestational age at delivery	37.9 ± 2.9	36.1 ± 2.9	38.1 ± 2.9	< 0.001
Birth weight	3122.4 ± 741.0	2683.9 ± 773.3	3174.3 ± 720.6	< 0.001
Male infant	268 (55.7)	20 (40.0)	248 (57.5)	0.02
^***a***^*p*-Values were calculated with Wilcoxon rank-sum test, chi-square test, or Fisher’s exact test where appropriate between preeclampsia status. ^***b***^*n* = 10 missing. ^***c***^*n* = 11 missing.				

Geometric mean [95% confidence interval (CI)] SG-corrected urinary BPA and phthalate metabolite concentrations by study visit are presented in Table 2. Variability of the phthalate metabolites during pregnancy in this population has been previously published by our group (Ferguson et al. 2014). Phthalate metabolites were detected in > 99% of samples except for MEHP (95.3%) and MCPP (96.8%), whereas total BPA was detected in 81.9% of samples. Geometric mean (95% CI) SG-corrected urinary BPA and phthalate metabolite concentrations by study visit and stratified by case/control status are presented in Table S1. Significant differences in SG-corrected phthalate levels by PE case–control status were detected for MECPP at visit 3 (61.1 vs. 36.4 ng/mL; *p* = 0.004). Levels of urinary SG were relatively constant across pregnancy with mean ± SD as follows for each visit and did not significantly differ between cases and controls: visit 1: 1.017 ± 0.008; visit 2: 1.014 ± 0.008; visit 3: 1.014 ± 0.008; and visit 4: 1.015 ± 0.007.

**Table 2:** Specific gravity–corrected urinary BPA and phthalate concentrations (ng/mL) [GM (95% CI)].

Analyte	Visit 1 (*n* = 479)	Visit 2 (*n* = 422)	Visit 3 (*n* = 412)	Visit 4 (*n* = 380)
BPA	1.34 (1.24, 1.45)	1.29 (1.20, 1.40)	1.39 (1.28, 1.51)	1.32 (1.22, 1.43)
MEHP	12.7 (11.3, 14.3)	11.3 (10.1, 12.7)	9.8 (8.8, 11.0)	9.9 (8.8, 11.3)
MEHHP	40.8 (36.3, 45.9)	34.1 (30.6, 38.1)	27.1 (24.1, 30.6)	34.8 (30.8, 39.3)
MEOHP	20.1 (17.9, 22.5)	18.2 (16.3, 20.2)	15.8 (14.0, 17.8)	20.1 (17.8, 22.6)
MECPP	51.8 (46.2, 58.0)	42.9 (38.4, 48.1)	38.5 (34.1, 43.4)*	48.7 (43.1, 55.1)
∑DEHP^*a*^	0.46 (0.41, 0.51)	0.39 (0.35, 0.43)	0.33 (0.30, 0.37)	0.41 (0.37, 0.46)
MBzP	6.9 (6.3, 7.7)	7.0 (6.2, 7.7)	6.9 (6.2, 7.7)	7.9 (7.0, 8.8)
MBP	17.9 (16.5, 19.5)	18.3 (16.7, 20.0)	17.4 (15.7, 19.2)	19.9 (18.3, 21.7)
MiBP	7.3 (6.8, 7.8)	7.2 (6.6, 7.8)	7.3 (6.7, 7.9)	9.0 (8.3, 9.8)
MEP	140.8 (123.2, 160.9)	146.5 (126.0, 170.4)	140.0 (120.6, 162.6)	147.4 (125.3, 173.4)
MCPP	2.3 (2.0, 2.5)	2.3 (2.0, 2.6)	1.9 (1.7, 2.2)	2.1 (1.9, 2.3)
GM, geometric mean. ^***a***^nmol/L. *p < 0.05 between cases and controls Wilcoxon rank-sum test.				

Adjusted hazard ratios (HR) and 95% CIs for onset of PE in association with an IQR increase in BPA and phthalate metabolite concentrations are presented in Table 3. Elevated HRs were observed in relation to an IQR increase in BPA concentration for visit 1 (HR = 1.53; 95% CI: 1.04, 2.25). In addition, there was a significant interaction with fetal sex (interaction term *p* = 0.005) where female fetuses had a greater risk for the mother developing preeclampsia compared with male fetuses. DEHP metabolites showed a consistent, significantly adverse association with preeclampsia. IQR increases in averaged MEHP (HR = 1.40; 95% CI: 1.03, 1.89) and ΣDEHP (HR = 1.79; 95% CI: 1.30, 2.46) levels were associated with a significant increase in the onset of preeclampsia. These adverse relationships were consistently observed at each time point (except time point 2). We also observed a consistent interaction with fetal sex with early (visit 1) and averaged DEHP metabolite levels where female fetuses were at greater risk for the mother developing preeclampsia (interaction term *p*-values ranging from 0.04 to 0.003). Interestingly, associations between an IQR increase in visit 4 DEHP metabolites and onset of preeclampsia were consistent, significant, and elevated compared with other time point biomarker levels, though due to the timing of the visit (median, 35.1 weeks), 50% (*n* = 25) of the preeclampsia cases had delivered before sample collection.

**Table 3:** Adjusted hazard ratios (95% CIs) for onset of preeclampsia in association with an interquartile range increase in BPA and phthalate metabolite concentrations (ng/mL).

Analyte	Average (visit 1–3) (50, 406)^*a*^	Visit 1 (50, 405)^*a*^	Visit 2 (42, 366)^*a*^	Visit 3 (44, 359)^*a*^	Visit 4^*b*^ (25, 341)^*a*^
BPA	1.14 (0.73, 1.79)	1.53 (1.04, 2.25)*	1.12 (0.61, 2.07)	0.68 (0.43, 1.07)	1.44 (0.80, 2.58)
MEHP	1.40 (1.03, 1.89)*	1.26 (0.97, 1.63)	1.14 (0.82, 1.60)	1.38 (1.02, 1.85)*	2.05 (1.35, 3.12)*
%MEHP	0.73 (0.52, 1.03)	0.75 (0.58, 0.97)*	0.90 (0.65, 1.25)	0.78 (0.61, 1.00)	1.17 (0.67, 2.03)
∑DEHP	1.79 (1.30, 2.46)*	1.52 (1.15, 2.00)*	1.24 (0.87, 1.75)	1.70 (1.24, 2.34)*	2.92 (1.61, 5.28)*
MBzP	0.93 (0.64, 1.35)	0.93 (0.65, 1.33)	1.08 (0.69, 1.70)	0.98 (0.63, 1.53)	1.83 (0.59, 5.65)
MBP	1.06 (0.74, 1.53)	1.14 (0.82, 1.56)	0.95 (0.58, 1.56)	1.09 (0.72, 1.65)	2.25 (0.98, 5.19)
MiBP	0.84 (0.58, 1.21)	1.22 (0.86, 1.74)	0.79 (0.49, 1.30)	0.64 (0.46, 0.90)*	1.54 (0.62, 3.82)
MEP	1.40 (1.00, 1.95)*	1.72 (1.28, 2.30)*	1.13 (0.76, 1.67)	1.15 (0.79, 1.68)	0.80 (0.46, 1.39)
MCPP	0.95 (0.71, 1.28)	1.07 (0.86, 1.33)	0.66 (0.45, 1.00)	1.52 (1.07, 2.15)*	2.37 (1.34, 4.18)*
Models were adjusted for specific gravity, maternal age, race, BMI, smoking during pregnancy, and infant sex. ^***a***^Cases, controls. ^***b***^If exposure measure occurred after preeclampsia diagnosis participants were removed from analysis (*n* = 6). *p < 0.05.					

Adjusted HRs stratified by infant sex are presented in Table 4. Consistent with our findings in our interaction models, among females, early exposure to BPA and DEHP metabolites resulted in a consistent and significant adverse relationship with onset of preeclampsia; among males this pattern was absent. Results were generally similar among female and male fetuses for lower-molecular-weight phthalates and exposure later in pregnancy to BPA or higher-molecular-weight phthalate metabolites. Caution should be exercised in interpreting results from this stratified population, given the low sample size.

**Table 4:** Adjusted hazard ratios (95% CIs) for onset of preeclampsia in association with an interquartile range increase in BPA and phthalate metabolite concentrations (ng/mL) stratified by infant sex.

	Average (visit 1–3)	Visit 1	Visit 2	Visit 3
Females
*n* (cases, controls)	(30, 170)	(30, 170)	(25, 156)	(27, 153)
BPA	1.37 (0.87, 2.16)	1.58 (1.20, 2.08)*	1.10 (0.77, 1.57)	0.81 (0.55, 1.17)
MEHP	1.67 (1.16, 2.42)*	1.55 (1.14, 2.12)*	1.28 (0.84, 1.94)	1.41 (0.98, 2.03)
%MEHP	0.84 (0.57, 1.24)	0.80 (0.61, 1.05)	0.79 (0.51, 1.23)	0.82 (0.64, 1.05)
∑DEHP	2.10 (1.44, 3.07)*	1.88 (1.34, 2.65)*	1.44 (0.94, 2.20)	1.75 (1.19, 2.58)*
MBzP	0.91 (0.57, 1.43)	1.00 (0.66, 1.51)	1.12 (0.62, 2.00)	0.82 (0.50, 1.36)
MBP	1.22 (0.71, 2.11)	1.35 (0.86, 2.11)	0.86 (0.39, 1.89)	1.16 (0.65, 2.10)
MiBP	0.68 (0.40, 1.14)	1.27 (0.85, 1.91)	0.41 (0.18, 0.92)*	0.59 (0.41, 0.85)*
MEP	1.44 (0.97, 2.15)	1.87 (1.32, 2.64)*	1.19 (0.75, 1.88)	1.14 (0.72, 1.81)
MCPP	0.96 (0.67, 1.36)	1.11 (0.87, 1.43)	0.56 (0.32, 0.98)*	1.58 (1.04, 2.42)*
Males
*n* (cases, controls)	(20, 236)	(20, 235)	(17, 210)	(17, 206)
BPA	0.67 (0.26, 1.71)	0.89 (0.38, 2.08)	1.37 (0.47, 3.97)	0.48 (0.18, 1.24)
MEHP	0.93 (0.52, 1.66)	0.84 (0.50, 1.42)	1.05 (0.57, 1.93)	1.24 (0.70, 2.20)
%MEHP	0.58 (0.37, 0.90)*	0.56 (0.34, 0.92)*	1.00 (0.66, 1.51)	0.69 (0.39, 1.23)
∑DEHP	1.21 (0.64, 2.26)	1.13 (0.66, 1.96)	1.05 (0.56, 1.96)	1.55 (0.84, 2.86)
MBzP	1.07 (0.55, 2.07)	0.96 (0.47, 1.95)	1.19 (0.55, 2.54)	1.55 (0.68, 3.51)
MBP	1.09 (0.63, 1.90)	1.09 (0.63, 1.89)	1.20 (0.65, 2.24)	1.13 (0.57, 2.23)
MiBP	1.23 (0.60, 2.52)	1.40 (0.65, 3.04)	1.88 (0.75, 4.69)	0.88 (0.38, 2.01)
MEP	1.32 (0.71, 2.47)	1.50 (0.88, 2.56)	1.05 (0.49, 2.27)	1.07 (0.52, 2.19)
MCPP	0.98 (0.54, 1.80)	1.07 (0.65, 1.75)	0.88 (0.50, 1.55)	1.43 (0.74, 2.76)
Models were adjusted for specific gravity, maternal age, race, BMI, and smoking during pregnancy. *p < 0.05.				

We further explored whether timing of disease onset—one method to delineate disease severity (earlier usually being more severe)—affected the associations. There were 8 (16.0%) cases of preeclampsia that were diagnosed < 34 weeks of gestation in this analysis, 23 (46.0%) that were diagnosed between 34 and 37 weeks, and 19 (38.0%) that were diagnosed > 37 weeks. Those diagnosed > 37 weeks had a consistent, significant relationship between higher DEHP metabolites or MEP and onset of PE (see Table S2). For those diagnosed between 34 and 37 weeks, higher BPA had a significant relationship with onset of PE. Interestingly, we observed no relationships between BPA or phthalate metabolites and onset of PE for those who were diagnosed < 34 weeks gestation.

As a sensitivity analysis, we additionally ran models after excluding cases of superimposed PE (*n* = 16), presented in Table S3. The previous association between BPA and PE found at visit 1 was no longer found to be significant. Conversely, our previously observed significant associations with DEHP metabolites, MEP, and MCPP all increased in strength.

## Discussion

In the present study of pregnant women in Boston we found urinary BPA and phthalate concentrations were significantly associated with onset of preeclampsia. After additional stratification by infant sex, we observed elevated HRs for females with early exposure in gestation (visit 1), in association with BPA and DEHP metabolite levels. Additionally, we observed this adverse relationship in those women who were diagnosed > 34 weeks of gestation. To our knowledge this is the first study to longitudinally assess BPA and phthalate concentrations in relation to onset of preeclampsia.

Preeclampsia is a multisystem, heterogeneous condition. Although diagnosis of preeclampsia is defined as new onset or worsening hypertension in the presence of significant proteinuria, neither of these conditions is specific to the pathophysiology of the disease (Rana et al. 2013).

It is generally agreed that the placenta (specifically trophoblast cells) is the root cause of this condition. Redman and Sargent (2005) broadly classified two categories of this disease: placental preeclampsia and maternal preeclampsia. In placental preeclampsia the placenta suffers from hypoxic conditions (arising from poor placentation) and undergoes extensive oxidative stress as pregnancy develops. Those with placental preeclampsia generally deliver earlier and manifest more severe symptoms of the disease. In maternal preeclampsia preexisting or underlying maternal conditions can lead to an abnormal maternal response to an otherwise normal pregnancy. These categories may arise independently or in combination. Additionally, Rana et al. (2013) have observed that profiles of angiogenic factors are related to preeclampsia and differences in these profiles may indicate similar categories to the disease that Redman and Sargent describe. Recently, Ferguson et al. (2015a) observed that DEHP metabolites and BPA were both related to increases in the ratio of sFlt-1 (soluble fms-like tyrosine kinase-1) to PLGF (placental growth factor), suggesting a plausible mechanistic link between increased exposure and heightened risk of placental preeclampsia. It is interesting to speculate that observations in this study not only support findings in Ferguson et al. (2015a) but may also suggest that these endocrine-disrupting chemicals are also related to maternal preeclampsia as well.

In a small cross-section study of women at delivery Leclerc et al. (2014) found elevated BPA concentrations in placentas, but not maternal or cord serum, of preeclamptic women versus normotensive controls. This same research group and others have also found that low BPA concentrations can induce apoptosis and inflammation in human trophoblastic cells *in vitro,* providing a potential mechanistic link of BPA to the pathophysiology of preeclampsia (Benachour and Aris 2009; Morice et al. 2011). Additionally, we observed that BPA exposure can alter angiogenic profiles, increasing the s-Flt/PlGF ratio, which Rana et al. (2013) have related to more severe versions of PE (Ferguson et al. 2015a). In our present study we found a significantly elevated hazard to developing preeclampsia in relation to visit 1 (median, gestational week 10.1) BPA levels, but this relationship was not observed with either averaged or other visit specific concentrations. Although it is plausible that timing of exposure to BPA may be critical for cellular damage to occur, albeit gestational week 6–12 coincides with significant trophoblastic cell remodeling, we cannot rule out the potential for chance findings at this time point. To our knowledge there has been no epidemiological study into the effects of phthalate exposure upon risk of preeclampsia.

In our subset analysis we observed a sex-specific susceptibility to BPA and phthalate exposure and onset of PE. Endocrine-disrupting effects of BPA and phthalates are well studied, and multiple animal and human studies have reported evidence of sex-specific adverse health effects resulting from exposure to either of these chemicals (Ashley-Martin et al. 2014; Braun et al. 2011; Cantonwine et al. 2015; Kubo et al. 2003). It has also been observed that there are sexually dimorphic responses with regard to placental function and placental disorders (Mao et al. 2010; Muralimanoharan et al. 2013; Osei-Kumah et al. 2011; Sood et al. 2006). Although our findings of sex-specific susceptibility to BPA/phthalate exposure and onset of PE are plausible, we acknowledge that with our limited sample size these may be chance findings.

Our study had several strengths, including a repeated time point assessment of BPA and phthalate exposure, ultrasound dating of gestational age, the ability to control for socioeconomic and clinical factors, and a physician panel to validate diagnosis and timing of onset of PE. Still, results from our secondary analyses of stratification upon infant sex, timing of disease onset, and visit 4 analysis should be interpreted in the context of the design, given that we were limited in our number of PE cases and are likely to be underpowered to detect subtle relationships. There was also no control for multiple comparisons, which may lead to an inflated type 1 error rate. We acknowledge that the few significant associations found in relation to BPA, MEP, and MCPP exposure may be attributable to chance alone and larger follow-up studies to replicate the findings are warranted. Although to our knowledge this was the first study of preeclampsia to use multiple urinary BPA and phthalate concentrations from each woman, the low temporal reliability of these concentrations across pregnancy may indicate that even with three to four repeated measures, there may still be substantial nondifferential exposure measurement error, which would further limit statistical power to detect associations.

## Conclusions

In conclusion, we found significant associations between urinary BPA and phthalate concentrations and onset of preeclampsia. We found consistent associations between DEHP metabolites and preeclampsia across pregnancy, with a potential heightened risk associated with concentrations later in pregnancy. With the relative lack of research into the impact of these ubiquitous endocrine-disrupting chemicals on placental function and risk of developing preeclampsia, this study highlights a critical need for future research.

## Supplemental Material

(123 KB) PDF
