# Supplementary material for: Urinary Concentrations of Bisphenol A and Phthalate Metabolites Measured during Pregnancy and Risk of Preeclampsia
Source: Environ Health Perspect. 2016 May 13;124(10):1651–5. doi: 10.1289/EHP188 (PMC5047771; doi:10.1289/EHP188)
Supplement: (123 KB) PDF [file EHP188.s001.acco.pdf]

Environ Health Perspect
DOI: 10.1289/EHP188

Note to readers with disabilities: *EHP* strives to ensure that all journal content is accessible to all readers. However, some figures and Supplemental Material published in *EHP* articles may not conform to 508 standards due to the complexity of the information being presented. If you need assistance accessing journal content, please contact ehp508@niehs.nih.gov. Our staff will work with you to assess and meet your accessibility needs within 3 working days.

Supplemental Material

Urinary Concentrations of Bisphenol A and Phthalate Metabolites Measured during Pregnancy and Risk of Preeclampsia

David E. Cantonwine, John D. Meeker, Kelly K. Ferguson, Bhramar Mukherjee, Russ Hauser, and Thomas F. McElrath

Table of Contents

Table S1. Specific gravity corrected urinary BPA and phthalate concentrations (ng/mL) geometric means (GM) and 95% Confidence Intervals (95% CI)

Table S2. Adjusted hazard ratios [95% confidence intervals] for onset of preeclampsia before 34 weeks of gestation in association with an interquartile range increase in BPA and phthalate metabolite concentrations (ng/mL)

Table S3. Adjusted hazard ratios [95% confidence intervals] for onset of preeclampsia in association with an interquartile range increase in BPA and phthalate metabolite concentrations (ng/mL) after removing cases of superimposed preeclampsia

Table S1. Specific gravity corrected urinary BPA and phthalate concentrations (ng/mL) geometric means (GM) and 95% Confidence Intervals (95% CI).

	Visit 1 (N=479)	Visit 2 (N=422)	Visit 3 (N=412)	Visit 4 (N=380)
	GM (95%CI)	GM (95%CI)	GM (95%CI)	GM (95%CI)
BPA				
Cases	1.56 [1.25, 1.94]	1.37 [1.10, 1.74]	1.39 [1.06, 1.81]	1.30 [0.97, 1.76]
Controls	1.32 [1.22, 1.43]	1.31 [1.21, 1.42]	1.38 [1.27, 1.51]	1.34 [1.23, 1.46]
MEHP				
Cases	12.0 [7.9, 18.2]	10.5 [6.9, 15.9]	10.4 [7.5, 14.4]	7.5 [4.9, 11.6]
Controls	12.8 [11.3, 14.5]	11.4 [10.1, 12.9]	9.8 [8.6, 11.0]	10.2 [8.9, 11.6]
MEHHP				
Cases	43.0 [29.4, 62.9]	34.4 [23.5, 50.2]	35.2 [24.1, 51.4]	32.9 [22.3, 48.3]
Controls	40.6 [35.9, 45.9]	34.1 [30.4, 38.2]	26.3 [23.2, 29.8]	35.0 [30.7, 39.8]
MEOHP				
Cases	21.8 [14.9, 31.9]	19.1 [13.1, 27.8]	21.4 [15.2, 30.1]	20.5 [14.4, 29.1]
Controls	19.9 [17.6, 22.5]	18.1 [16.2, 20.2]	15.2 [13.4, 17.3]	20.0 [17.6, 22.7]
MECPP				
Cases	68.0 [45.9, 100.7]	52.6 [36.0, 76.8]	61.1 [43.2, 86.4]*	60.5 [40.3, 91.1]
Controls	50.1 [44.5, 56.4]	42.0 [37.3, 47.2]	36.4 [32.0, 41.4]	47.8 [42.0, 54.4]
∑DEHP[a]				
Cases	0.53 [0.37, 0.78]	0.42 [0.30, 0.61]	0.46 [0.33, 0.64]	0.45 [0.31, 0.64]
Controls	0.45 [0.40, 0.50]	0.38 [0.34, 0.43]	0.32 [0.28, 0.36]	0.41 [0.36, 0.46]
MBzP				
Cases	6.7 [4.9, 9.1]	8.0 [5.6, 11.4]	7.4 [5.7, 9.5]	8.2 [5.9, 11.4]
Controls	7.0 [6.3, 7.8]	6.8 [6.1, 7.7]	6.8 [6.1, 7.7]	7.8 [7.0, 8.8]
MBP				
Cases	17.7 [14.1, 22.3]	19.5 [15.2, 25.0]	18.7 [15.6, 22.3]	21.3 [16.9, 26.9]
Controls	18.0 [16.4, 19.7]	18.1 [16.4, 20.0]	17.2 [15.4, 19.2]	19.8 [18.0, 21.6]
MiBP				
Cases	7.4 [5.8, 9.3]	6.9 [5.5, 8.6]	6.5 [5.5, 7.8]	8.7 [7.1, 10.6]
Controls	7.3 [6.7, 7.8]	7.2 [6.6, 7.9]	7.4 [6.8, 8.1]	9.1 [8.3, 9.9]
MEP				
Cases	193.3 [122.4, 305.1]	170.2 [105.9, 273.5]	173.5 [103.0, 292.4]	237.0 [128.8, 435.9]
Controls	135.7 [118.0, 156.0]	144.1 [122.7, 169.1]	136.5 [116.8, 159.5]	141.3 [119.4, 167.3]
MCPP				
Cases	2.4 [1.9, 3.1]	1.8 [1.4, 2.4]	2.4 [1.7, 3.2]	2.2 [1.6, 3.1]
Controls	2.3 [2.0, 2.5]	2.4 [2.1, 2.7]	1.9 [1.7, 2.1]	2.1 [1.9, 2.4]

*p-value < 0.05 between cases and controls Wilcoxon Rank Sum test

[a] nmol/L

Table S2. Adjusted hazard ratios [95% confidence intervals] for onset of preeclampsia before 34 weeks of gestation in association with an interquartile range increase in BPA and phthalate metabolite concentrations (ng/mL)

	Average (Visit 1-3)	Visit 1	Visit 2	Visit 3
< 34 weeks				
N (cases, controls)	(8, 432)	(8, 431)	(8, 379)	(6, 368)
BPA	0.81 [0.19, 3.48]	2.63 [0.95, 7.29]	0.88 [0.15, 5.32]	0.25 [0.07, 0.88]*
MEHP	0.57 [0.22, 1.43]	0.74 [0.29, 1.85]	0.49 [0.17, 1.37]	0.61 [0.26, 1.43]
MEHHP	0.88 [0.31, 2.49]	0.93 [0.43, 2.02]	0.55 [0.17, 1.75]	1.29 [0.50, 3.29]
MEOHP	0.68 [0.24, 1.96]	0.84 [0.38, 1.83]	0.44 [0.14, 1.38]	1.06 [0.44, 2.59]
MECPP	1.02 [0.35, 2.95]	1.04 [0.40, 2.69]	0.65 [0.23, 1.85]	1.32 [0.51, 3.41]
∑DEHP	0.88 [0.31, 2.50]	0.97 [0.39, 2.39]	0.57 [0.18, 1.76]	1.16 [0.44, 3.04]
MBzP	0.61 [0.21, 1.83]	0.56 [0.20, 1.60]	0.78 [0.22, 2.75]	0.68 [0.21, 2.21]
MBP	0.35 [0.10, 1.25]	0.49 [0.20, 1.25]	0.48 [0.11, 2.03]	0.63 [0.24, 1.63]
MiBP	0.50 [0.24, 1.04]	0.57 [0.23, 1.40]	0.39 [0.14, 1.10]	0.63 [0.27, 1.46]
MEP	0.62 [0.22, 1.70]	0.59 [0.24, 1.48]	0.88 [0.29, 2.71]	0.62 [0.22, 1.73]
MCPP	0.80 [0.29, 2.20]	0.93 [0.43, 2.00]	0.70 [0.24, 2.00]	1.29 [0.49, 3.38]
≥ 34 and < 37 weeks				
N (cases, controls)	(23, 432)	(23, 429)	(19, 379)	(21, 368)
BPA	2.06 [1.03, 4.13]*	1.64 [0.86, 3.14]	1.97 [0.69, 5.61]	1.40 [0.74, 2.65]
MEHP	0.97 [0.55, 1.72]	0.91 [0.53, 1.56]	0.98 [0.52, 1.83]	1.16 [0.70, 1.91]
MEHHP	1.18 [0.63, 2.20]	1.03 [0.61, 1.76]	1.09 [0.55, 2.13]	1.44 [0.83, 2.49]
MEOHP	1.36 [0.72, 2.59]	1.11 [0.63, 1.96]	1.23 [0.64, 2.34]	1.51 [0.88, 2.60]
MECPP	1.96 [1.09, 3.52]*	1.65 [0.93, 2.93]	1.60 [0.90, 2.85]	1.78 [1.05, 3.02]*
∑DEHP	1.53 [0.85, 2.76]	1.29 [0.74, 2.24]	1.38 [0.74, 2.57]	1.59 [0.93, 2.69]
MBzP	1.18 [0.65, 2.15]	1.31 [0.69, 2.46]	1.31 [0.63, 2.73]	1.27 [0.65, 2.46]
MBP	1.18 [0.67, 2.06]	1.36 [0.79, 2.34]	1.34 [0.69, 2.63]	1.14 [0.61, 2.14]
MiBP	0.78 [0.42, 1.47]	1.32 [0.65, 2.66]	0.93 [0.38, 2.26]	0.66 [0.38, 1.16]
MEP	1.47 [0.82, 2.65]	1.61 [0.93, 2.79]	1.23 [0.61, 2.47]	1.74 [0.91, 3.32]
MCPP	0.97 [0.54, 1.75]	1.18 [0.75, 1.83]	0.77 [0.39, 1.51]	1.24 [0.70, 2.20]
≥ 37 weeks				
N (cases, controls)	(19, 432)	(19, 431)	(16, 379)	(17, 368)
BPA	0.78 [0.44, 1.41]	1.17 [0.70, 1.96]	0.59 [0.26, 1.31]	0.74 [0.43, 1.28]
MEHP	1.35 [0.94, 1.96]	1.25 [0.91, 1.73]	1.12 [0.75, 1.67]	1.17 [0.82, 1.67]
MEHHP	1.33 [0.87, 2.03]	1.32 [0.96, 1.80]	0.93 [0.59, 1.47]	1.22 [0.81, 1.82]
MEOHP	1.83 [1.19, 2.80]*	1.50 [1.07, 2.09]*	1.14 [0.74, 1.76]	1.56 [1.06, 2.30]*
MECPP	2.35 [1.59, 3.48]*	1.83 [1.28, 2.63]*	1.31 [0.88, 1.95]	2.00 [1.37, 2.91]*
∑DEHP	1.86 [1.25, 2.74]*	1.55 [1.11, 2.16]*	1.13 [0.73, 1.72]	1.63 [1.12, 2.38]*
MBzP	1.03 [0.68, 1.57]	0.85 [0.57, 1.29]	1.15 [0.70, 1.90]	1.19 [0.75, 1.88]
MBP	1.31 [0.90, 1.91]	1.11 [0.74, 1.68]	1.10 [0.64, 1.89]	1.66 [1.13, 2.45]*

MiBP	1.20 [0.76, 1.88]	1.19 [0.76, 1.86]	1.07 [0.60, 1.93]	1.19 [0.72, 1.97]
MEP	1.49 [1.00, 2.23]*	1.78 [1.26, 2.53]*	1.24 [0.78, 1.96]	1.15 [0.71, 1.85]
MCPP	0.99 [0.68, 1.46]	1.15 [0.88, 1.51]	0.56 [0.34, 0.92]*	1.39 [0.94, 2.08]

Models adjusted for specific gravity.

* statistically significant (p<0.05)

Table S3. Adjusted hazard ratios [95% confidence intervals] for onset of preeclampsia in association with an interquartile range increase in BPA and phthalate metabolite concentrations (ng/mL) after removing cases of superimposed preeclampsia.

Analyte	Average (Visit 1-3) (34, 406)	Visit 1 (34, 405)	Visit 2 (28, 366)	Visit 3 (30, 359)	Visit 4[a] (24, 341)
BPA	0.83 [0.48, 1.43]	1.23 [0.77, 1.96]	0.94 [0.45, 1.94]	0.58 [0.33, 1.00]	1.30 [0.60, 2.81]
MEHP	1.64 [1.17, 2.30]*	1.36 [1.02, 1.81]*	1.22 [0.84, 1.78]	1.73 [1.24, 2.42]*	2.54 [1.59, 4.06]*
MEHHP	1.22 [0.83, 1.81]	1.18 [0.88, 1.58]	0.85 [0.55, 1.30]	1.43 [0.97, 2.11]	3.38 [1.81, 6.29]*
MEOHP	1.68 [1.13, 2.49]*	1.35 [0.98, 1.85]	1.13 [0.75, 1.70]	1.85 [1.27, 2.70]*	3.92 [2.00, 7.68]*
MECPP	2.46 [1.68, 3.59]*	1.86 [1.32, 2.62]*	1.52 [1.05, 2.21]*	2.42 [1.66, 3.51]*	3.29 [1.73, 6.24]*
∑DEHP	1.84 [1.28, 2.65]*	1.48 [1.08, 2.03]*	1.25 [0.84, 1.85]	2.02 [1.40, 2.91]*	3.66 [1.92, 6.98]*
MBzP	0.91 [0.59, 1.40]	0.86 [0.57, 1.28]	0.98 [0.58, 1.65]	1.08 [0.64, 1.79]	2.95 [1.15, 7.61]*
MBP	1.23 [0.83, 1.82]	1.19 [0.82, 1.72]	0.99 [0.56, 1.76]	1.49 [0.98, 2.25]	2.28 [1.07, 4.89]*
MiBP	0.83 [0.55, 1.26]	1.17 [0.78, 1.76]	0.80 [0.45, 1.44]	0.65 [0.43, 0.96]*	1.88 [0.56, 6.33]
MEP	1.66 [1.15, 2.40]*	2.16 [1.55, 3.02]*	1.21 [0.80, 1.84]	1.39 [0.90, 2.14]	0.76 [0.45, 1.29]
MCPP	0.95 [0.67, 1.33]	1.09 [0.85, 1.40]	0.62 [0.39, 1.00]	1.53 [1.01, 2.29]*	3.22 [1.83, 5.65]*

Models adjusted for specific gravity, maternal age, race, BMI, smoking during pregnancy, and infant sex

[a] If exposure measure occurred after preeclampsia diagnosis participants were removed from analysis (N=6)

* statistically significant ($p<0.05$)
